# A Sport-Based Intervention to Increase Uptake of Voluntary Medical Male Circumcision Among Adolescent Male Students: Results From the MCUTS 2 Cluster-Randomized Trial in Bulawayo, Zimbabwe

**DOI:** 10.1097/QAI.0000000000001046

**Published:** 2016-10-06

**Authors:** Zachary A. Kaufman, Jeff DeCelles, Kenneth Bhauti, Rebecca B. Hershow, Helen A. Weiss, Cynthia Chaibva, Netsai Moyo, Fennie Mantula, Karin Hatzold, David A. Ross

**Affiliations:** * Department of Infectious Disease Epidemiology, Faculty of Epidemiology and Population Health, MRC Tropical Epidemiology Group, London School of Hygiene & Tropical Medicine, London, United Kingdom;; † Department of Health Behavior, University of North Carolina-Chapel Hill Gillings School of Global Public Health, Chapel Hill, NC;; ‡ Department of Health Policy & Management, University of North Carolina, Chapel Hill, NC;; § Grassroot Soccer Zimbabwe, Bulawayo, Zimbabwe;; ‖ Department of Nursing and Midwifery Sciences, National University of Science and Technology, Bulawayo, Zimbabwe; and; ¶ Population Services International Zimbabwe, Harare, Zimbabwe.

**Keywords:** male circumcision, demand creation, adolescents, HIV prevention

## Abstract

Mathematical models suggest that 570,000 HIV infections could be averted between 2011 and 2025 in Zimbabwe if the country reaches 80% voluntary medical male circumcision (VMMC) coverage among 15- to 49-year-old male subjects. Yet national coverage remains well below this target, and there is a need to evaluate interventions to increase the uptake.

**Methods:**

A cluster-randomized trial was conducted to assess the effectiveness of Make-The-Cut-Plus (MTC+), a single, 60-minute, sport-based intervention to increase VMMC uptake targeting secondary school boys (14–20 years). Twenty-six schools in Bulawayo, Zimbabwe, were randomized to either receive MTC+ at the start (intervention) or end (control) of a 4-month period (March to June 2014). VMMC uptake over these 4 months was measured via probabilistic matching of participants in the trial database (n = 1226 male participants; age, 14–20 years; median age, 16.2 years) and the registers in Bulawayo's 2 free VMMC clinics (n = 5713), using 8 identifying variables.

**Results:**

There was strong evidence that the MTC+ intervention increased the odds of VMMC uptake by approximately 2.5 fold (odds ratio = 2.53; 95% confidence interval, 1.21 to 5.30). Restricting to participants who did not report being already circumcised at baseline, MTC+ increased VMMC uptake by 7.6% (12.2% vs 4.6%, odds ratio = 2.65; 95% confidence interval, 1.19 to 5.86). Sensitivity analyses related to the probabilistic matching did not change these findings substantively. The number of participants who would need to be exposed to the demand creation intervention to yield one additional VMMC client was 22.7 (or 13.2 reporting not already being circumcised). This translated to approximately US dollars 49 per additional VMMC client.

**Conclusions:**

The MTC+ intervention was an effective and cost-effective strategy for increasing VMMC uptake among school-going adolescent male subjects in Bulawayo.

## Abstract 2

Supplemental Digital Content is Available in the Text.

## INTRODUCTION

Randomized controlled trials have shown that voluntary medical male circumcision (VMMC) reduces female-to-male HIV transmission by 50%–60%.^1–3^ Mathematical models have estimated that more than 3.3 million new HIV infections could be averted in 2011–2015 if VMMC coverage targets are reached.^4^ This includes 570,000 infections averted in Zimbabwe (almost half of the projected new infections) if the country achieves a target of 80% coverage of 15- to 49-year-old male subjects.^4^ Despite the resources and efforts invested in VMMC scale-up, progress in the 14 priority countries has been slower than anticipated.^5,6^ Zimbabwe is falling short of its target of 80% VMMC coverage by 2015, having reached just 31% of the target by December 2014,^6–8^ underlining the urgent need to identify and scale-up effective, demand-creation interventions.

UNAIDS and the World Health Organization have stressed the importance and urgency of increasing VMMC uptake among adolescent male subjects, identifying schools and sports as 2 possible vehicles for intervention.^9^ A 2013 systematic review of sport-based HIV prevention interventions found promising evidence of effectiveness, although few studies had assessed effects on the uptake of HIV-related services and none on VMMC.^10^

In 2014, free VMMC services in Bulawayo were only available at 2 clinics run by Population Services International Zimbabwe: The Bulawayo Eye Clinic and the Lobengula Clinic. These clinics have been operating since 2009 and 2012, respectively, and completed 26,383 VMMC procedures in 2012.

A recent cluster-randomized trial measured the effectiveness of Make The Cut (MTC), a 60-minute soccer-based intervention aiming to increase VMMC uptake among Zimbabwean men aged 18–50 years who were members of soccer teams in Bulawayo, Zimbabwe. Enrolling 47 soccer teams and 736 men, the Male Circumcision Uptake Through Soccer (MCUTS) trial found that the MTC intervention increased VMMC uptake almost 10-fold [4.8% uptake among uncircumcised intervention participants, compared with 0.5% among control participants; odds ratio (OR), 9.81; 95% confidence interval (CI), 0.93 to 103.2; *P* = 0.06].^11^ Both quantitative and qualitative findings from MCUTS suggested that MTC's acceptability was higher among younger men (18–20 years) participating in the trial. The objective of the present study was to assess the effectiveness of a modified intervention (Make-The-Cut-Plus or MTC+) focused on this age group, among male students attending secondary schools in Bulawayo.

## METHODS

### Trial Setting

The trial was conducted from March to October 2014 in Bulawayo, Zimbabwe. Thirty secondary schools in the Bulawayo district with more than 50 male students enrolled in Forms 3–5 were invited to participate. The trial had originally intended to randomize 22 schools. In April 2014, however, the number of schools was increased to 26 after it became clear that the average number of study participants per school would be lower than expected. Ethical approval for the study was obtained from the Interventions Research Ethics Committee of the Medical Research Council of Zimbabwe and the London School of Hygiene and Tropical Medicine.

### Participants

The study enrolled male students attending Forms 3–5 (age, 14–20 years) in the 26 trial secondary schools in Bulawayo. All male participants in these grades at selected schools were eligible to participate in the study, including those who were circumcised.

### Intervention

The MTC+ intervention consisted of a 60-minute session delivered in schools between 04/03/2014 and 10/06/2014 by a trained facilitator known as a “Coach”—a circumcised man aged 18–30 years, trained by Grassroot Soccer.

The MTC+ session consists of an interactive game, a personal story shared by the Coach, and a group discussion. The game uses the popularity of soccer among Zimbabwean males to initiate discussions on a potentially sensitive topic, an approach shown to improve HIV-related knowledge, attitudes, and behaviors.^10^ During the interactive and educational soccer penalty shoot-out (see Supplemental Digital Content A, http://links.lww.com/QAI/A824), the goalkeeper metaphorically tries to protect himself from HIV infection. In the first round, the goalkeeper represents an uncircumcised man who does not use condoms, frequently failing to stop the ball. In the next round, after participants identify that VMMC can reduce the HIV risk of the goalkeeper, the goal's width is reduced, representing the partial protection offered by VMMC. In the final round, 4 additional defenders help block the reduced-size goal, representing the additional protection of consistently and correctly using condoms in addition to VMMC. Key messages communicated during the activity focus on the health benefits of VMMC, including improved hygiene and protection from sexually transmitted infections.

In line with social learning theory,^12^ our theory of change posits that circumcised coaches can teach VMMC information and, through sharing their own experiences, build participants' self-efficacy to undergo VMMC through personal stories. Coaches build personal connections with participants and address barriers to VMMC, such as fear of pain during and after the surgery and perceived benefits, such as decreased risk of HIV infection and improved hygiene. These personal stories help coaches facilitate discussions with participants on their own perceived barriers and enablers. The intervention's design is explained in a separate article.^13^

In the week following intervention delivery, Coaches follow-up with participants who express interest in receiving VMMC at the end of the interpersonal session via phone call, SMS (short message service), or Whatsapp (mobile phone-based messaging platform). Coaches schedule a meeting with a group of participants who, after going through MTC+, had said that they wanted to receive VMMC. Transport to the clinic is provided for the group.

Participants at the first 5 intervention schools were offered a nonmonetary incentive (t-shirt or soccer match ticket) valued at approximately $5 if they went for VMMC. The intention had been to offer incentives to all intervention participants, but the incentive component was removed in April 2014 at the request of the Population Services International because of concerns that it could be unsustainable or potentially coercive. As a result, 5 intervention schools received the offer of the incentives, whereas the other 8 did not. Control schools were provided the intervention (not including incentives) at the end of the trial.

### Outcomes

The primary outcome was VMMC uptake from 07/03/2014 to 06/07/2014, as determined by probabilistic matching between the trial's participant database and the registers from the 2 VMMC clinics in Bulawayo. Linkage was carried out using Linkage Wiz software, which has been widely used for linkage of health and patient data.^14^ Eight identifying variables included in the study consent forms and clinic registers were weighted based on relative likelihoods of correctly identifying a match (Table 1). Each potential pair was scored, earning points for exact or partial matches, and losing points for disagreements between variables. Partial matches were defined as 1–2 character differences in string variables (eg, name, street address, or phone number) or a 2–3 year age difference.

**TABLE 1.**
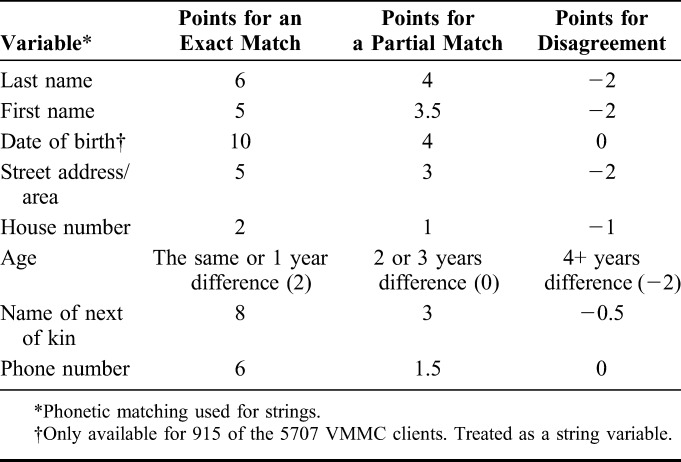
Variables and Weights Used for Probabilistic Matching

Links were classified as definite matches (18.00+ points), probable matches (14.00–17.99), possible matches (12.00–13.99 points), or nonmatches (11.99 or fewer). To prevent false-positives and false-negatives, possible and probable matches were manually reviewed by 2 investigators (just the database records, no medical information), liaising where they did not agree (2 of the 24 reviewed matches) to come to an agreement. The primary outcome used to measure VMMC uptake included definite matches and 18 probable/possible matches classified as links after manual review. Sensitivity analyses were performed using only computed probabilistic scores, respectively restricting the outcome definition to (1) definite, probable, or possible matches, (2) definite or probable matches, and (3) only definite matches.

### Randomization

Schools were the unit of randomization. To maximize group comparability, before randomization, schools were divided into 3 strata based on student enrollment data: private schools, large public schools (≥300 students enrolled), and small public schools (<300 students). Within each stratum, schools were randomized in a 1:1 ratio at a public event held in Bulawayo. For each stratum, each school's name was written on a piece of paper. Papers were then blindly drawn from a concealed bucket to assign them to the intervention or control group.

### Sample Size

Assuming an average of 80 male participants per school, 20% baseline circumcision prevalence,^15^ VMMC uptake of 2% in the control group over 4 months, and coefficient of variation (*k*) = 0.3, we originally estimated that a sample size of 11 intervention and 11 control schools would provide more than 90% power to detect a 3-fold increase in VMMC uptake because of the intervention (*P* < 0.05).^16^ Three weeks into the trial, it became apparent that baseline reported circumcision prevalence would likely be higher than 20%, and the mean number of participants per school, even with several repeat visits, was closer to 50. To retain power of 90%, we enrolled 4 additional schools (2 small public and 2 large public), allocating them using the same randomization method.

### Data Collection

Before and after intervention (follow-up), participants completed a 20-minute questionnaire, assessing demographic information, socioeconomic status, VMMC-related knowledge and perceptions, reported circumcision status, and reported sexual behavior. This questionnaire was administered confidentially on touch screen mobile phones using Open Data Kit Collect. This method had previously been used with high acceptability with adolescents in South Africa and adult men in Bulawayo.^17^

Daily registers from the Bulawayo Eye Clinic and the Lobengula Clinic were photographed, and data were transcribed with double entry into an electronic database. The data in these registers had been handwritten during each client's visit by clinic receptionists.

### Statistical Methods

Primary analyses were carried out by intention-to-treat using random-effects logistic regression, adjusting for school-level clustering and school type. Three levels of sensitivity analysis were carried out, as described above. Each analysis was performed (1) across all participants and (2) as a “restricted analysis,” restricted to participants not reporting being circumcised at baseline. A prespecified subgroup analyses was carried out between predefined age groups, as was a post-hoc analysis between participants who were offered or not offered incentives. Age was considered an *a priori* effect modifier. Assessment of effect modification by age was carried out using a likelihood ratio test. A prespecified, secondary, cluster-level analysis was carried out considering the small number of clusters. Cost-effectiveness calculations were carried out by dividing the number of additional VMMC clients generated by the intervention (based on its absolute effectiveness) by the total cost of the intervention.

### Masking

Because of the nature of the intervention, it was not possible to mask participants' or Coaches' to the schools' group allocation. However, clinic receptionists were masked to clients' trial participation. Probabilistic linkage and review of probable/possible links were done blind to school and study group.

## RESULTS

### Recruitment

**FIGURE 1.**
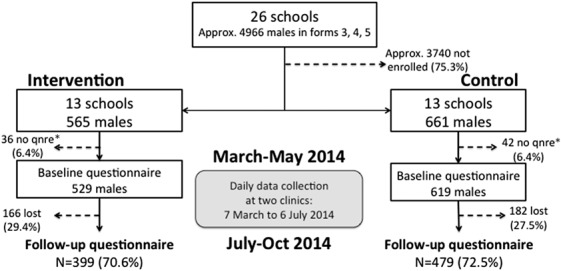
Flow diagram of study participants. *qnre, questionnaire.

Figure 1 shows the flow of schools and participants in the trial. Twenty-six schools were enrolled, averaging 368 students in forms 3–5 per school (according to reported enrollment numbers). Based on 2010 national enrollment estimates from the Zimbabwe Ministry of Education for forms 3–5,^18^ we estimate that 52% of these reportedly enrolled students were male, making the estimated potential study population approximately 4966 males. Of these, 1226 were enrolled in the trial (all enrolled participants under 18 years obtained written parental consent). All the enrolled participants in the intervention arm received the MTC+ intervention. Of the 1226 enrolled, 78 (6.4%) did not complete baseline questionnaires, leaving 529 intervention and 619 control participants with baseline questionnaire completed between 10/03/2014 and 09/05/2014. After roughly 4 months, 399 (70.6%) intervention and 479 (72.5%) participants completed end line questionnaires between 07/07/2014 and 13/10/2014.

There was good balance in baseline characteristics by trial arm (Table 2). Most participants (88.1% intervention, 90.5% control) attended public schools. At baseline, approximately half (49.4% intervention and 47.2% control) self-reported being circumcised, and approximately one-third reported having a circumcised father (34.1% intervention, 33.9% control). Median age in both arms was 16.2 years.

**TABLE 2.**
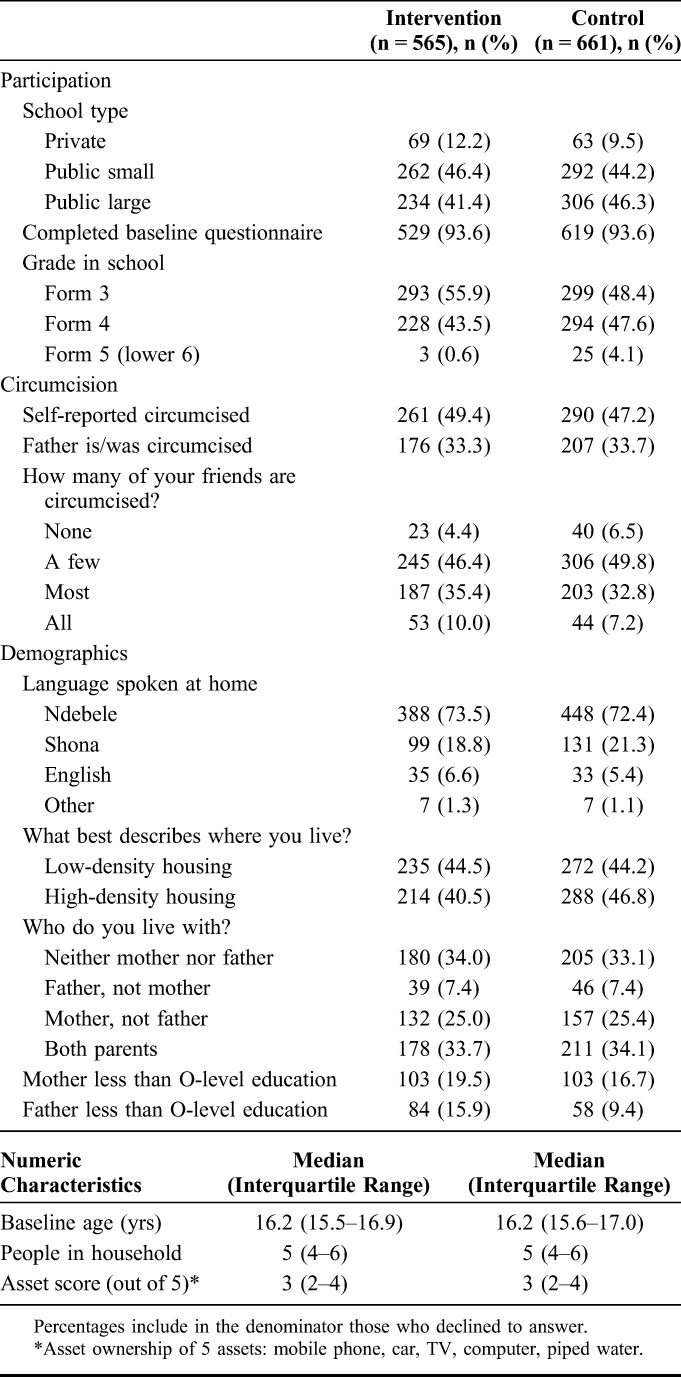
Baseline Sample Characteristics by Study Group (n = 1226)

### Effect of the MTC+ Intervention on VMMC Uptake

Overall, 41 intervention participants (7.3%) and 19 control participants (2.9%) were circumcised during trial follow-up (OR, 2.46; 95% CI, 1.20 to 5.04; *P* = 0.01; Table 3). Results were similar when restricted to participants not reporting being already circumcised at baseline (12.2% vs 4.6%; OR, 2.60; 95% CI, 1.22 to 5.55; *P* = 0.01). Based on these results, the number needed to be exposed to the intervention to yield 1 additional VMMC client was 13 participants not reporting already being circumcised at the baseline survey or 23 total participants.

**TABLE 3.**
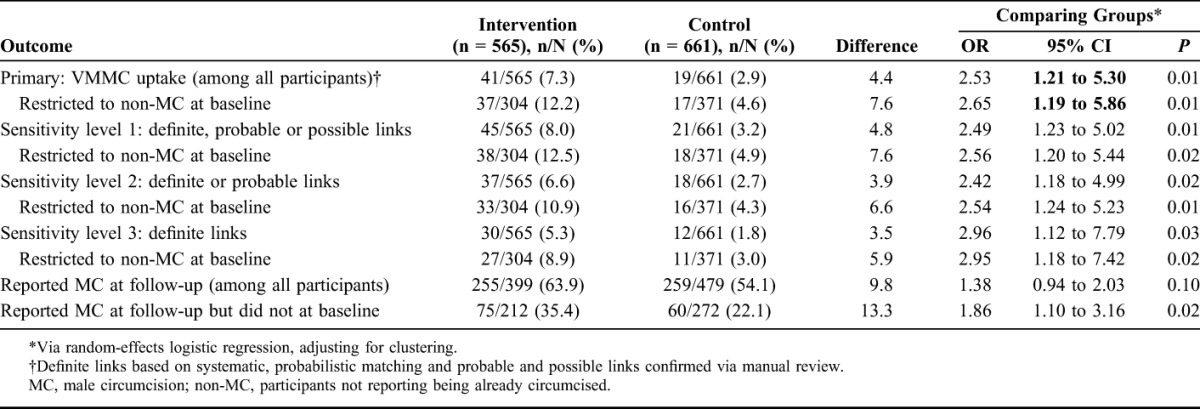
VMMC Uptake by Study Group, Including Sensitivity Analyses (n = 1226)

Sensitivity analyses did not change these findings substantively. Results remained consistent when considering all participants and when restricting to those not reporting being already circumcised at baseline (Table 3). Secondary cluster-level analyses also yielded similar results.

In the follow-up questionnaire, 255 intervention (63.9%) and 259 control participants (54.1%) self-reported being circumcised. Restricting to those not reporting being already circumcised at baseline, there was strong evidence that a higher proportion of intervention participants (35.4%) than control participants (22.1%) reported having been circumcised at follow-up but not at baseline (OR, 1.86; 95% CI, 1.10 to 3.16; *P* = 0.02).

### Subgroup Analyses

Table 4 presents subgroup analyses by age group. In the control arm, VMMC uptake increased with age (2.0% among 14- to 15-year olds vs 9.3% among 18- to 20-year olds; OR_trend_, 2.15; 95% CI, 0.98 to 4.77). The intervention effects were similar among 14- to 15-year olds (OR, 2.97; 95% CI, 0.97 to 9.14) and 16- to 17-year olds (OR, 3.32; 95% CI, 1.50 to 7.31), but there was little evidence of an intervention effect among 18- to 20-year olds (OR, 1.45; 95% CI, 0.32 to 6.50). There was little evidence of effect modification by age group (*P* = 0.47).

**TABLE 4.**
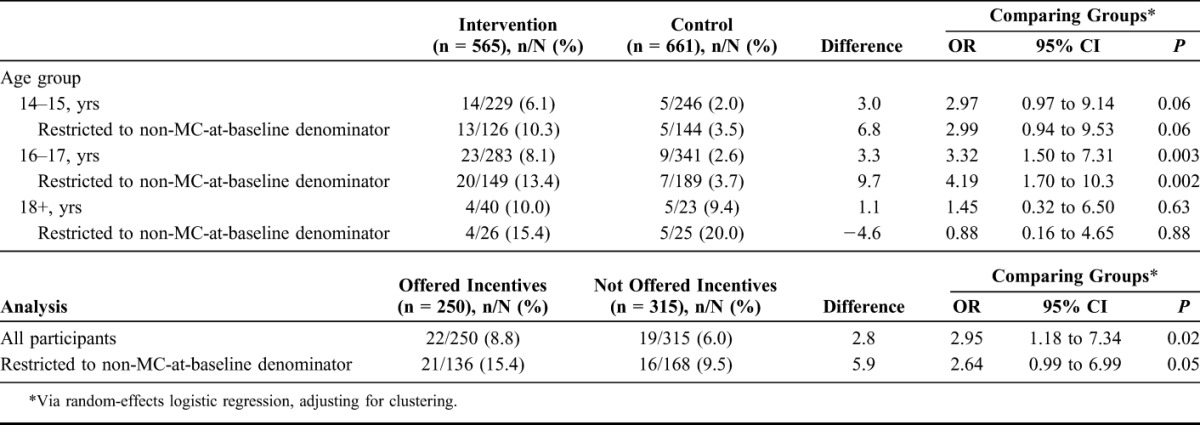
Subgroup Analysis of VMMC Uptake by Age Group and Whether Schools Were Offered Incentives

Odds of uptake were nearly 3 times higher among intervention participants offered the incentives (8.8%) than among those who were not (6.0%) (OR, 2.95; 95% CI, 1.18 to 3.27; *P* = 0.02). A similar effect was observed in the restricted analysis (OR, 2.65; 95% CI, 0.99 to 6.99; *P* = 0.05).

### Cost-Effectiveness

Implementing MTC+ with 565 intervention participants cost a total of $1121.83 or $1.99 per participant. The total cost of the intervention included training workshop for coaches; transportation for coaches; materials including a t-shirt, printed curriculum, soccer balls, cones, and laminated cards; coach stipend of $3.50 per half-day of work; and 15% overhead. Forty-one of these participants went for VMMC, resulting in a cost of $27.36 per client in the intervention arm. The approximate cost per additional VMMC client was $45.31 among all participants or $48.61 among participants not reporting being circumcised at baseline.

## DISCUSSION

In this trial, MTC+ increased VMMC uptake among adolescent males in Bulawayo schools over a 4-month period by approximately 7.6% or roughly 2.5-fold. The observed effect was consistent whether considering all participants or restricting the analysis to participants not reporting being already circumcised at baseline. It was also consistent across 3 levels of sensitivity analysis, with the strictest level of analysis suggesting that the relative effect may be closer to 3-fold. Although one should treat sensitive self-reported data from adolescents with caution,^19^ it was encouraging that strong evidence of effectiveness was also observed when assessing self-reported VMMC uptake at follow-up. The absolute and relative effects observed were similar to those observed for the middle-tier intervention group ($8.75) in a 2013 trial assessing conditional economic compensation for VMMC uptake among men (25–49 years) in Kenya.^20^

There was evidence of increased uptake of VMMC with age in both arms, but no evidence that the intervention effect differed by age. The removal of incentives in the middle of the trial unintentionally allowed for a nonrandomized experiment to understand the role of nonmonetary incentives in increasing uptake. While bearing limitations, our subgroup analysis findings supported previous evidence that small incentives can be effective in increasing VMMC demand.

Almost half the participants at baseline reported being circumcised. This was higher than expected and suggests that other adolescent-targeted VMMC demand creation interventions in Bulawayo have had some effect, either in getting students to go for circumcision or in increasing the desirability bias in reporting or both. If the former, it also suggests that those participants getting circumcised in the trial were relatively late adopters of VMMC. If introduced into a setting where most early adopters had not yet gone for VMMC, it is possible that MTC+ would have greater effectiveness and cost-effectiveness.

The trial had limitations. First, only a quarter of reportedly enrolled male students in the schools participated in the trial, which would affect external validity if those who chose not to participate differed by circumcision status or willingness to become circumcised. Second, the estimated absolute increase in VMMC uptake may be over- or underestimated depending on the validity of the self-reported circumcision status of the participants at baseline. However, the relative effect estimates for the unrestricted and restricted analyses were consistent, suggesting any bias introduced from restriction was nondifferential. Third, the identifier-based linkage process was imperfect, but it was not logistically feasible to use biometrics for participant linkage. The robust probabilistic linkage approach used was the next best alternative. Another possible limitation was that the study only captured VMMC patients at the 2 study clinics. It is possible some participants underwent VMMC at a private clinic or outside Bulawayo during the trial period. The period of observation for VMMC uptake at the 2 study clinics was relatively short (4 months). If the intervention led participants to go for VMMC later, the trial would have missed these links. The trial had approximately 28% loss-to-follow-up between baseline and end line surveys, although this did not differ by arm and would have only influenced questionnaire findings. The trial was not designed to measure the effect of different components of the intervention—interpersonal educational session, coach accompaniment to the clinic, and transportation—perhaps limiting our ability to conclude which components are crucial to VMMC uptake. However, a process evaluation was conducted to investigate the perceived value of each component. Finally, poor handwriting by students on consent forms and/or by receptionists on clinic registers may have resulted in imperfect capture of identifying information. This, in turn, may have yielded false-negatives in the linkage process. The use of phonetic matching and fuzzy logic reduced the risk of false-negatives resulting from poor handwriting or inconsistent spelling. The masked, manual, post-linkage review further reduced the risk of false-negatives and false-positives.

Given the urgent need to increase VMMC uptake in Zimbabwe and other countries with generalized HIV epidemics, it is crucial to take effective interventions to scale to prevent new infections. This study provides strong evidence of the effectiveness of MTC+ in Bulawayo schools. Notably, the trial was only carried out in one city, so the results should be treated with cautious optimism when considering the potential impact of MTC+ at scale across Zimbabwe and/or other countries. If the effectiveness remains consistent at scale, the MTC+ intervention could generate substantial new VMMC demand among adolescent males in schools. However, this brief, low-cost intervention would not be sufficient on its own to achieve the national VMMC target of 80% coverage. Thus, there remains a need for additional effective interventions—especially those targeting adult males—potentially including mass media, interpersonal communication interventions, and interventions using monetary or nonmonetary incentives.
